# Solventless Crosslinking of Chitosan, Xanthan, and Locust Bean Gum Networks Functionalized with β-Cyclodextrin

**DOI:** 10.3390/gels6040051

**Published:** 2020-12-15

**Authors:** Max Petitjean, Florian Aussant, Ainara Vergara, José Ramón Isasi

**Affiliations:** Department of Chemistry, University of Navarra, 31080 Pamplona, Spain; mpetitjean@alumni.unav.es (M.P.); faussant@alumni.unav.es (F.A.); avergara.3@alumni.unav.es (A.V.)

**Keywords:** cyclodextrin polymers, chitosan, xanthan gum, locust bean gum, green synthesis, sorption, hydrogels

## Abstract

The incorporation of cyclodextrins into polymeric crosslinked gels of hydrophilic nature can be useful for promoting the sorption of hydrophobic molecules and/or modulating the release of active principles. The covalent addition of these excipients to the matrix integrates their solubilizing effect that can contribute to increase the capacity of retention of hydrophobic substances. In this study, three diverse polysaccharides, chitosan, xanthan gum, and locust bean gum, were crosslinked with or without β-cyclodextrin, using citric acid in different ratios, to create hydrogel matrices. Through a green synthetic path, the efficient production of soluble and insoluble (hydrogel) networks functionalized with β-cyclodextrin was achieved by means of a solventless procedure. The characterization of their chemical composition, swelling in water, and their sorption and release behavior were also carried out in this work.

## 1. Introduction

Polysaccharides are long chained carbohydrates mostly synthesized by Nature. This versatile family includes diverse macromolecules, depending on their origins, their chemical structure or functional groups. Xanthan gum (XG) is a polysaccharide synthesized by *Xanthomonas campestris* bacteria, composed of a D-glucose skeleton and a branch every two-monomer units formed by one glucuronic acid between two mannoses. An acid pyruvate function is present at the end of this arm, on the last mannose unit [1]. As the xanthan gum, locust bean gum (LBG) is a branched polysaccharide, made by a primary mannose chain, with a crosslinked galactose every four monomers. Both can be used without modifications as thickeners in the food industry for example [2]. Chitosan (CS) is a linear polysaccharide, derivative from chitin, constituted of glucosamine and N-acetyl-glucosamine units. Thanks to its bioactive and biocompatibility properties, it can be used in medical devices [3].

Cyclodextrins are a family of cyclic oligosaccharides composed of six or more glucose units, being that with seven units, named β-cyclodextrin, the most common one. Used by themselves as carriers because of their complexation power with hydrophobic molecules, cyclodextrins can easily be crosslinked to form soluble or insoluble polymer networks [4]. Thus, cyclodextrin nanosponges can be created following different paths, with diverse crosslinking agents, going from simple matrices to imprinted cyclodextrin polymers [5].

Polysaccharides, and more specifically, food polysaccharides [6,7], can also be crosslinked, either physically or chemically [8,9,10], to yield versatile matrices susceptible of being used as drug carriers [11], probiotic delivery systems [12], release systems for agrochemicals [13] or in food packaging [14], adsorbents in wastewater treatment [15], and even obtain ‘ordered’ hydrogels with additional potential capabilities [16]. Since it has been shown that most crosslinkers, such as the commonly used glutaraldehyde, can produce cytotoxic effects [10], poly(carboxylic acids) have been proposed instead. Citric acid is a well-known ‘green’ crosslinking agent, allowing the creation of bridges between polysaccharides [17], cyclodextrins [18] and both types of carbohydrates at the same time [19]. The degradability of this type of crosslinking constitutes an additional advantage in the search for sustainable crosslinked polymers [20]. In addition, the carboxylic acid added by the citric bridge confers a negative charge to the polymer, which allows the sorption of different molecules such as heavy metals and cationic species [21].

The thermochemical reaction of citric acid with starch and other polysaccharides has been studied previously [22]. In the present work, we investigate the solventless crosslinking of chitosan, xanthan gum and locust bean gum with citric acid using β-cyclodextrin to functionalize the polysaccharide networks and provide them with its encapsulation capabilities. The resulting hydrogel matrices could compete with other cyclodextrin polymers prepared using harmful reagents and costly procedures.

## 2. Results and Discussion

### 2.1. Selection of the Crosslinking Process Conditions

In the absence of a suitable solvent, such as water in the case of most polysaccharides, the crosslinking reactions will occur if time and temperature are high enough to permit an adequate diffusion and thermal energy for the process. In order to ascertain the best conditions to achieve an optimal yield of both polysaccharide crosslinking and cyclodextrin functionalization, temperatures between 140 and 180 °C were tested in the first place, using the same weight ratio for the three reagents (i.e., citric acid, β-cyclodextrin and each polysaccharide). Then, once an optimal temperature was fixed at 170 °C (see below), the reaction times were varied between 10 and 120 min. The same procedures were applied to the three diverse polysaccharides selected for this study: xanthan gum (XG), locust bean gum (LBG), and a 90% deacetylated chitosan (CS) (see structures in Scheme S1, Supplementary Materials).

**Figure 1:**
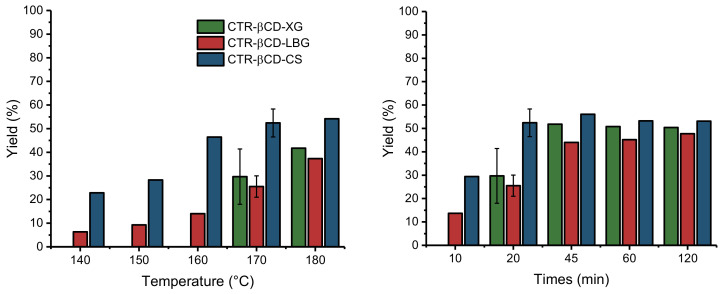
Yield of insoluble crosslinked fraction of xanthan gum (XG), locust bean gum (LBG) and chitosan (CS) with β-cyclodextrin and citric acid: processed for 20 min at different temperatures (**left**) and processed at 170 °C for different times (**right**). (For the yield of the soluble crosslinked fractions, see Supplementary Materials, Figure S1). (Error bars obtained using *n* = 3 for those samples).

As can be seen in Figure 1, for the three polysaccharides used, both temperature and time influence the reaction yield (expressed as the percent of insoluble product obtained after the whole process). For every polysaccharide, a similar maximum yield was found: 55% in the case of chitosan, 50% for xanthan and 45% for locust bean gum. Those are reached for temperatures above 170 °C and for reaction times of at least 20 min.

In every experiment, chitosan crosslinking processes show a higher yield, which could be explained by its additional capacity to react with itself thanks to the Maillard reaction [23]. The noticeable browning of these chitosan resins (see Supplementary Materials, Tables S1 and S2) is a clear indication that such a crosslinking is taking place, as will be confirmed using FTIR (see below). In addition to this, amino groups of chitosan can also react with citric acid to produce amide crosslinking bridges, although esterification is certainly more probable considering the relative amounts of hydroxyl and amine groups present.

The esterification reaction of citric acid with xanthan gum does not yield an insoluble fraction for temperatures below 170 °C. For this limit temperature, a reaction time of 10 min is not enough to reach the gel point either. It becomes evident that, in the absence of other types of crosslinking, reaching the melting point of citric acid is a necessary condition. Moreover, in addition to its fusion, a sufficient time is required to diffuse inside the saccharide networks. Once the reaction temperature was set at 170 °C, it was observed that the maximum yield is achieved in 45 min (in contrast to 20 min for the chitosan networks). For a fixed reaction time (20 min), the yield will obviously increase with the reaction temperature. It is expected that higher temperatures and times will produce the degradation of the reagents, as some browning of the products also indicate (Table S2), so the process was not carried out beyond the described limits. For locust bean gum networks, insoluble fractions were collected in all cases, with a higher yield for high temperature and long reaction times.

Even though the molten citric acid can easily diffuse within the polysaccharide powdered solids, the reaction with β-cyclodextrin (which melts above the onset of its decomposition at 250 °C) is more difficult. As will be shown below when analyzing the reaction byproducts (Section 2.4), the amount of unreacted β-cyclodextrin collected in the soluble fraction, after the washing process, can be around 30% in the case of 1:1 cyclodextrin/polysaccharide mixtures (see Table S3). The highest found (above 50%) corresponds to the network prepared with no polysaccharide (i.e., citric acid plus cyclodextrin).

The esterification reactions in aqueous solutions are commonly catalyzed using acids or bases. Solventless esterification processes of carbohydrates also use phosphates [4] or hypophosphites [24]. The influence of the selected catalyst, disodium phosphate (Na_2_HPO_4_), was assessed by comparing the results obtained using two other common catalysts (also buffering additives and raising agents in the food industry), namely potassium pyrophosphate (K_4_P_2_O_7_) and sodium hydrogen carbonate (NaHCO_3_), using the same mole ratios. Some differences are found after this modification. For instance, with pyrophosphate, the yield decreases for CS networks although it is higher for the other two polysaccharides. In the case of NaHCO_3_, the influence is lower: the same yield for CS, lower for XG, and intermediate for LBG (see results in Figure S2).

In addition, the mole ratio of disodium phosphate was also varied in a different set of experiments, in order to ascertain its effect. The yield for CS and XG matrices is ca. 10% lower when an excess of Na_2_HPO_4_ is added (a smaller effect is found in the case of LBG networks). In fact, a sample prepared with no catalyst was also successfully crosslinked. Thus, it can be concluded that the presence of a catalyst is not required at these process conditions (170 °C for 20 min). Nevertheless, some differences in the bonding mechanism can be described with the aid of FTIR spectroscopic results (Figure S3).

### 2.2. Composition of the Crosslinked Networks

Infrared spectroscopy is a very convenient tool to analyze the products of the crosslinking process, provided that we can select suitable bands that are representative of the components. In this respect, the glucose units that form the cyclodextrin moieties do not present any unique vibration modes that are absent in the polysaccharides, so it becomes difficult to ascertain, with precision, the cyclodextrin proportion in the polyester networks. Nevertheless, the amount of accessible cyclodextrin cavities, which constitutes its unique feature, can be assessed by other indirect spectroscopic methods (see below, Section 2.3.3).

**Figure 2:**
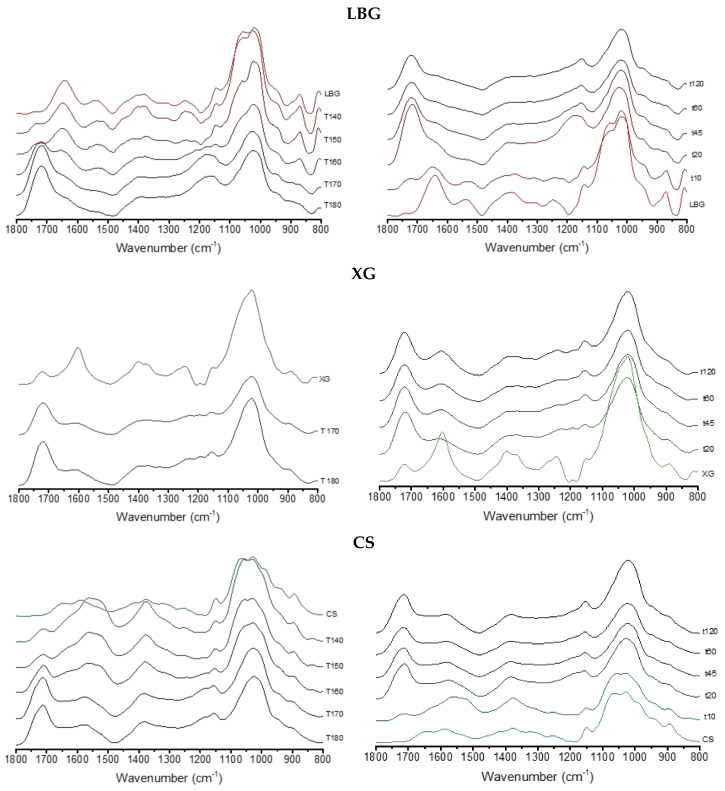
Infrared spectra in the 1800–600 cm^−1^ region of pure LBG, XG and CS, and the products of the crosslinking reaction with β-CD and citric acid at different temperatures (**left**) and times (**right**).

In addition to the common carbohydrate infrared bands, locust bean gum also possesses two characteristic bands located at 1741 and 1537 cm^−1^, which are attributed to protein impurities (ca. 5%) from the seed that remain after processing [2]. In Figure 2, the progress of the crosslinking reaction with citric acid can be assessed by the advent and growth of bands at 1750 and 1716 cm^−1^, corresponding to the carbonyl stretching modes of esters and acids groups, respectively. For both series of experiments, i.e., varying reaction time or temperature, these two bands increase, as expected, showing the evolution of the crosslinking process.

In the case of xanthan gum, the presence of carboxylic acid groups in its structure complicates the analysis of the 1800–1500 cm^−1^ spectral region. The pure polysaccharide shows two peaks here: a more intense one located at 1600 cm^−1^, corresponding to the carboxylate groups, and a less intense one at ca. 1725 cm^−1^ due to its acid groups. For the xanthan/cyclodextrin networks, the new carbonyl band at 1740 cm^−1^, characteristic of the crosslinked ester groups, overcomes the vibration mode at that location and increases in the same way as for LBG crosslinked products (see Figure 2).

Infrared spectra corresponding to the chitosan/cyclodextrin networks are somewhat different. The reaction between protein amine groups and the carbonyl groups of reducing sugars at temperatures between 140 and 160 °C, known as the Maillard reaction, is responsible for the browning of cooked meat or breads. The chitosan polymers crosslinked with citric acid show a brownish color, whose intensity correlates well with both the increasing temperatures and times of reaction (see Supporting Information, Tables S1 and S2). This is an indication of the production of colored products by the reaction between chitosan amine groups and saccharide carbonyls present in the mixture. In contrast, the crosslinked products obtained with either LBG or XG present much paler tinges that are especially evident for the highest reaction times; in these cases, this is an indication of the onset of dehydration degradation products. Interestingly, for the lower temperatures and times chosen for this study, the Maillard-like reactions will be the main responsible ones for the formation of crosslinking bridges in the case of the chitosan networks. The infrared bands located at 1523 and 1570 cm^−1^, corresponding to secondary and primary amides respectively, are intense for these polymers (see Figure 2). Nevertheless, for higher temperatures and reaction times, the carbonyl bands at 1743 and 1700 cm^−1^ overcome those amide bands. Thus, there are two mechanisms of the crosslinking process for the chitosan networks, and their relative importance is a function of the reaction temperature. To summarize, different crosslinked functions are present in chitosan matrices. The reaction temperature and time allow us to modify the proportion of these functions. Thus, a resin prepared at low temperature or time will present a crosslinking made mostly by the Maillard reaction, while a product made at a higher temperature or for a longer time will be mostly crosslinked by esterification.

Finally, the influence of the catalyst selected will also be much better analyzed with the aid of infrared spectroscopy. The carbonyl region of the spectra corresponding to chitosan/cyclodextrin insoluble crosslinked networks are not identical, and the main differences are found in the amide 1570 cm^−1^ band. In contrast, the ester carbonyl stretching bands show very similar shapes, irrespective of the catalyst used. The largest “Maillard” amide bands correspond to the use of an excess of phosphate (twice the standard amount) or the use of pyrophosphate, while hydrogen carbonate does not seem to influence the result (see Figure S3).

### 2.3. Properties of the Insoluble Matrices

#### 2.3.1. Stability against Hydrolysis

Several parameters will be of interest in case these novel materials are intended to be used as cyclodextrin-functionalized hydrogel sorbents. In the first place, the citrate crosslinks are hydrolysable, which can be either an advantage or a drawback, depending on the desired application. The ester groups can be broken at acidic or basic pHs, but there can be other covalent attachments, produced by secondary reactions taking place at high temperatures, that are not easily detached and produce a more ‘permanent’ crosslinking.

In this work, the saponification process was carried out at room temperature using concentrated sodium carbonate (see Section Materials and Methods). This solution (pH ca. 12) is capable of completely hydrolyzing the crosslinked β-cyclodextrin (with no polysaccharide in the formulation). In contrast, the resins prepared using polysaccharides are not fully solubilized, a clear indication that a small amount of crosslinking unions other than ester groups must be present (in fact, attempts using stronger basic solutions and higher temperatures proved unsuccessful as well).

A remarkable decrease in the percentage of non-saponifiable residue is observed for LBG polymers, as the synthesis temperature is increased (from 55% of residue when the synthesis temperature is 140 °C, down to 25% at 180 °C). In xanthan gum insoluble polymers, the percentage of non-saponifiable residue is close to 50%. Chitosan polymers show a very similar ratio of non-hydrolysable residue, around 60%. On the other hand, for those samples prepared at 170 °C using different reaction times, a steady (although not too pronounced) increase of the non-saponifiable amount is observed as the reaction time is increased (with the exception of the shortest reaction time for LBG). The infrared spectra (see Supplementary Materials, Figure S4) show, as expected, the loss of citrate groups in all cases, which permits the characteristic polysaccharide modes that appear in the same area to be clearly observed again. (For the highest reaction times and temperatures, a small band, attributable to citric acid groups, is perceived, which indicates that the saponification has not been effective enough to fragment all the bonds in these samples).

#### 2.3.2. Swelling of Insoluble Functionalized Polysaccharide Matrices

**Figure 3:**
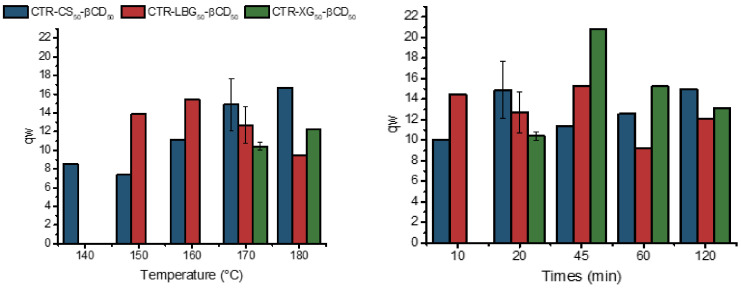
Swelling mass ratios for LBG, XG, and CS crosslinked hydrogels prepared using a 50/50 CD/PS feed ratio at different temperatures (**left**) and for different times at 170 °C (**right**). (Error bars obtained using *n* = 3 for selected samples.)

The hydrophilic nature of the citrate crosslinked polysaccharide gel networks is reflected in their corresponding swelling capacities. The water molecules within hydrogels can be bound either strongly or weakly to the networks, or non-bound, and the distribution of the types of binding is linked to the properties of the hydrogel [25]. A proper degree of swelling will facilitate the diffusion of sorbate molecules suitable to be entrapped either in the networks themselves or, depending on their characteristics, included in the cyclodextrin hydrophobic cavities. The degrees of swelling measured are different depending on how they are defined, whether in volume or in mass. The former are affected by the interstitial space between particles, which is somewhat problematic when the dry samples are in powdered form, as in this case. If the mass ratio swelling degrees are considered (i.e., mass of swollen gel over mass of dry gel), the CD-functionalized polysaccharide networks prepared in this work show values between 8 and 20, which corresponds to moderate to high values of swelling power far from the superabsorbent characteristics of other materials (Figure 3). The highly hydrophilic nature of these gels can be confirmed by studying their swelling behavior in other sorbents (Table S4). Neither 1-octanol nor acetone produce a significant swelling in any of these crosslinked resins.

Obviously, these degrees of swelling are a function of the chemical affinity of the polymeric structures, but they also greatly depend on the crosslinking density. In the case of chitosan/cyclodextrin gels synthesized at 170 °C, the observed values are quite similar, irrespective of the reaction time. In contrast, there is a steady two-fold increase on the swelling as the reaction temperature is increased from 140–150 °C up to 180 °C. At the lower reaction temperatures, the amount of citrate groups present is remarkably lower (see above), which will have an effect on the network hydrophilicity, and the crosslinking is mainly due to Maillard crosslinking. 

In contrast, locust bean gum networks show the opposite swelling behavior with respect to the synthesis temperature. The swelling decreases for the highest temperatures tested. If the evolution of the citrate band is observed, it can be concluded that, in this case, the higher crosslinking density is responsible for the increase in the compactness of the networks, which hinders the excessive swelling. When analyzing the results obtained for a constant temperature of synthesis as a function of time, a moderate decrease in the degree of swelling is also detected. 

Finally, the swelling behavior for the xanthan gum networks is more difficult to analyze due to the lack of data for the lower synthesis temperatures. Nevertheless, for those prepared at 170 °C, it is observed that the degree of swelling reaches a maximum for a 45 min reaction and then decreases again when the crosslinking density is continuously increasing.

#### 2.3.3. Sorption Properties of the Crosslinked Networks

**Figure 4:**
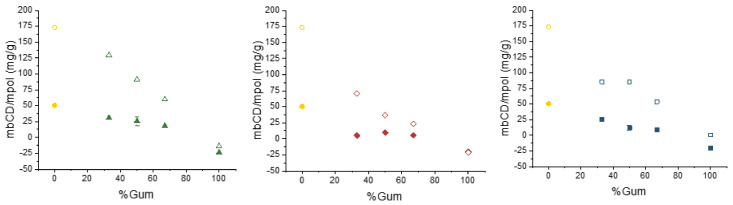
Amount of available cyclodextrin (mg/g) as a function of the cyclodextrin/gum hydrogel composition for xanthan gum (**left**), locust bean gum (**center**) and chitosan (**right**). Filled symbols, insoluble fractions; empty symbols, soluble fractions. (Error bars for 50% insoluble fractions obtained with *n* = 5 experiments.)

The third characteristic of interest of these novel materials, once their stability against hydrolysis and their degrees of swelling have been considered, is their ability to entrap hydrophobic molecules. Several model sorbates have been selected for this study. First of all, phenolphthalein has been proposed, due to its specificity, as an appropriate probe to ascertain the amount of β-cyclodextrin cavity sites available for inclusional interactions [26,27]. In addition to the 1:1 weight ratio mixtures of β-cyclodextrin and the corresponding polysaccharides, two other ratios (2:1 and 1:2) have been prepared for comparison purposes. All samples share the same amount of citric acid, catalyst, synthesis temperature and time. It was observed that the amount of available cyclodextrin sites in the insoluble crosslinked fractions (measured as mg/g, see Figure 4) were not high, and depended, as expected, on the cyclodextrin ratio. When comparing the three polysaccharides, xanthan gum available cyclodextrin values are higher than those of chitosan, while the latter are in turn higher than the locust bean gum ones. This result will be confirmed when the soluble fractions are analyzed (see below, Section 2.4). Notice that, for the crosslinked matrices prepared with no cyclodextrin, this method produce ‘negative’ (although small) values for the estimated cyclodextrin content, which indicates that the polysaccharides might have some effect in the results.

In addition, our previous studies showed that 1-naphthol is capable of interacting also with the cyclodextrin cavities because of the size and polarity of its naphthalene ring, while, at the same time, it can establish specific interactions with the hydrophilic networks via hydrogen bonding. The results obtained for the sorption of the resins prepared at different times and temperatures of synthesis are not conclusive, but they point to the fact that LBG matrices are better sorbents for this particular model molecule. The amounts sorbed for the chitosan and xanthan gum matrices functionalized with cyclodextrin are not too different though to those obtained for “pure” cyclodextrin (i.e., CD/citric acid) matrices. It becomes evident that some synergistic behavior must be responsible for the much better sorption observed in the case of LBG/CD matrices. In fact, as can be observed in Figure S5, pure LBG matrices (with no cyclodextrin) do absorb more 1-naphthol than pure chitosan and pure xanthan gum ones, but this value is significantly lower than that of the LBG/CD matrix.

**Figure 5:**
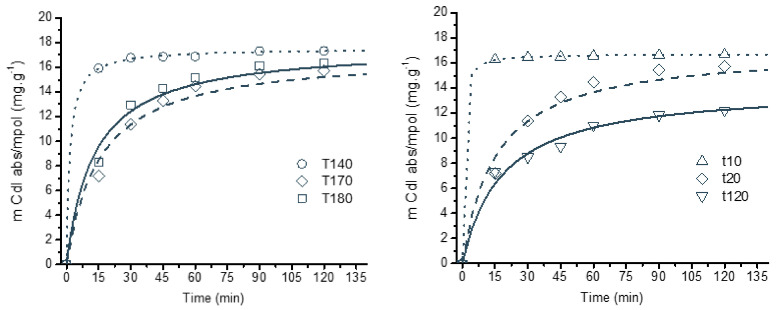
Sorption kinetics of indigo carmine (measured in mg absorbed per gram of resin) for the chitosan matrices prepared at three different temperatures (**left**) and at the same temperature (170 °C) for different times (**right**). (Lines represent curve fittings using a pseudo second order model, Table S5).

The sorption of indigo carmine, an anionic dye, was also tested. As expected, only chitosan/cyclodextrin matrices absorbed a significant amount of this model molecule via interionic interactions. Interestingly, the temperature of synthesis of the CS/CD polymers did not affect the amount absorbed, while a clear decreasing trend was observed for the ones prepared using higher reaction times (Figure 5). A kinetic study of the sorption process of indigo carmine in these matrices has shown that the process is much faster for the low reaction temperatures or short reaction times samples, i.e., for matrices crosslinked via the Maillard reactions.

Lastly, in order to understand the influence of each polysaccharide in the matrix, the sorption behavior for a mixture prepared with two dyes, a cationic one, methylene blue (MB), and anionic methyl orange (MO), was analyzed [28]. The absorption of cationic MB is similar for the four samples and considerably higher than the absorption of MO in all cases. The contribution of the abundant free acid groups seems to be most relevant in the sorption of MB dye. On the other hand, for the anionic MO, significant differences are found: the chitosan matrix absorbs 23% of the initial quantity when the three others absorb around 10% (Table S6). Now, the chitosan amine groups explain this absorption as that of indigo carmine (see above). Moreover, the dried matrices were placed in pure water to study the liberation of the loaded dyes. All the networks release the MO dye but keep MB molecules trapped inside (the release curves are shown in Figure S6). The chitosan matrix releases more MO, in absolute terms (although it amounts 30% of the total loaded). For the xanthan matrix, 60% is released, while the two others lose about 40% of their initial capacity.

### 2.4. Characterization of the Soluble Gel Fractions

**Figure 6:**
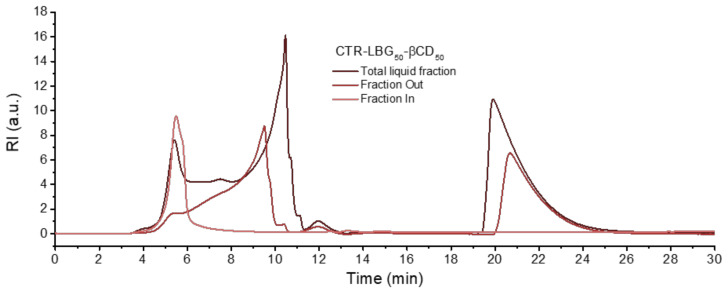
SEC results of soluble fraction obtained from LBG/CD (1:1 ratio) reaction product: total liquid fraction before dialysis, dialyzed liquid (“fraction out”) and high molecular weight fraction retained inside the 3.5 kDa dialysis membranes (“fraction in”).

Soluble crosslinked resins based on cyclodextrins have also been studied [4], as they can provide some interesting applications. Nevertheless, the main goal of this investigation was to optimize the process conditions to yield the maximum amount of crosslinked resin suitable for sorption processes. Thus, the soluble fractions recovered after washing and filtrating the resins were also analyzed for comparison purposes. These washed-out liquid fractions were concentrated, dialyzed through 3.5 kDa membranes and lyophilized. The pore size of these membranes does not allow the polysaccharide long chains to exit so they will be recovered in the lyophilized product. Size-exclusion chromatographic analyses of the soluble fractions before and after dialysis showed a high molecular weight peak on the exclusion limit of the column, plus two additional peaks: unreacted β-cyclodextrin molecules (which, interestingly, appear at times beyond the permeation limit of the column), and a small molecular weight byproduct fraction that appears at intermediate times. The latter is probably a mixture of unreacted citric acid, catalyst, and citrate modified β-cyclodextrin units, which is not of interest although there is a considerable amount of it (see Figure 6 for a LBG/CD 1:1 matrix and Figure S7 for the other polysaccharides). The area of the unreacted β-cyclodextrin was measured and, using a calibration standard, the corresponding percentage on the basis of the reagent mixture was calculated as discussed earlier (see also Supplementary Materials, Table S3). Values ca. 20–30% are found, which is a considerable amount, taking into account that there will also be some citrate-functionalized cyclodextrin units that appear at the intermediate region and do not constitute a ‘successful’ product either.

The yields of the purified soluble fractions are quite low, compared to those of the insoluble product. The results can be found in Figure S1 and they range between 3 and 10% of the initial weight with some values even lower than that.

Once the soluble crosslinked polysaccharide has been separated from the liquid fractions, an estimation of its size using the SEC results is not possible. As mentioned above, these molecules are too large and appear at the exclusion limit, which in this column corresponds to a polyethylene oxide standard of 10^6^ Da. Dynamic light scattering is a more appropriate technique instead. The results (see Table S7, Supplementary Materials) show, as expected, the large size and heterogeneity of the crosslinked networks. The hydrodynamic radii values of the LBG/CD networks are considerably higher than those of the parent pure LBG sample (up to ten-fold its value). In the case of CS/CD two of the produced networks show similar sizes to that of the parent chitosan sample, while one of them is somewhat bigger. Finally, XG/CD networks seem to be smaller than the parent xanthan gum, which points to a possible aggregation of the latter.

After the approximate sizes of the soluble fractions are known, some additional work is needed to study the composition of these networks. Infrared spectroscopy permits to compare their citrate contents with those of the insoluble ones, while the phenolphthalein discoloration method will allow us ascertain whether the available cyclodextrin sites are more abundant in the case of the soluble fractions. Finally, the interactions of the networks with 1-naphthol will be analyzed using fluorescence spectroscopy.

With regard to the 1800–1500 cm^−1^ region of the infrared spectra (see Figure S8, Supplementary Materials), the soluble LBG/CD polymers show different citrate bands for the three CD/LBG ratios measured. In contrast, the 1710 cm^−1^ bands for the corresponding insoluble fractions are more similar. The soluble xanthan gum networks also present significantly different intensities on their citrate bands, in contrast to the chitosan ones. It becomes evident that the amounts of citrate groups are, in all cases, relatively higher for the soluble fractions (see Figure S8).

In order to ascertain whether these soluble polysaccharide chains have also been successfully functionalized with cyclodextrin, the phenolphthalein assay was also carried out for them. Figure 4 shows that, in comparison to the corresponding insoluble fractions, the soluble ones possess a much higher ratio of accessible cyclodextrin cavities. The highest difference is found for the xanthan gum polymers, while those made with locust bean gum present more similar values. Moreover, the amount of available cyclodextrin seems to correlate with the ratio of citrate groups found by infrared analysis (shown in Figure S8).

As in the case of the insoluble matrices, the interactions of the soluble networks with 1-naphthol were analyzed. (The fluorescence quenching analysis of the soluble polysaccharides is presented in Section Supplementary Materials.) As can be seen in Table S8, among the pure polysaccharides, LBG possesses the highest interaction constant value with 1-naphthol, followed by xanthan gum and chitosan. On the other hand, in the case of the crosslinked samples, LBG/CD, CS/CD, and ‘pure’ crosslinked CD show similar values, while XG/CD presents the smaller interaction constant. Interestingly, locust bean gum seems to establish the best interactions with this particular sorbate, in good agreement with the sorption results shown above for the insoluble resins.

## 3. Conclusions

The aim of this study was to determine the optimal conditions for the solventless crosslinking of xanthan gum, locust bean gum, and chitosan, functionalized with β-cylodextrin, and to analyze the characteristics of these matrices and their suitability as sorbents. The highest reaction yields found for high temperatures (170–180 °C) are due to a greater crosslinking extent between citric acid and the saccharide chains. The 1500–1800 cm^−1^ infrared region is useful to ascertain the composition of both soluble and insoluble fractions. Besides the abundant acid and ester groups, responsible for the crosslinking reactions, chitosan also binds to sugar molecules through Maillard-type reactions. In general, the soluble crosslinked polymers present a higher ratio of ester groups. The sorption of a model molecule such as 1-naphthol in the insoluble networks demonstrates its affinity for the CD-rich matrices, but especially for those prepared with locust bean gum, which show a much more pronounced synergistic effect when compared to the ‘pure’ CD crosslinked matrix. The binding of 1-naphthol to soluble polymer fractions has confirmed this.

The obtained results are of interest for the design of novel ‘green’ sorbents prepared following a facile procedure. The weak point of such a simple method is the homogeneization of the solid mixtures, an important concern especially if a scale-up of the process is intended. A possible solution to this drawback is to prepare a paste by kneading the mixtures using a small amount of water. Other polysaccharides and their mixtures could be used for a proper tailoring design of the cyclodextrin functionalized matrices suitable for a specific sorbate. In addition, a physical modification of the networks by adding porogen agents could also improve their capabilities. The rheological properties of such matrices, including compactness and stability of the gels, could be important for some applications, such as the filtering of solutions with substances of interest. The swelling behaviors, related to chemical composition and degree of crosslinking, are also very different for all these matrices. Thus, their suitability for potential applications such as the sorption and release of bioactive phenolic compounds, the cleaning of wastewaters or the valorization of agricultural wastes need to be tested as well.

## 4. Materials and Methods

### 4.1. Materials

Anhydrous citric acid (CTR) (99.5%) was purchased from Panreac AppliChem (Barcelona, Spain). Xanthan gum (XG) (lot #SLBG3388V) and locust bean gum (LBG) (lot #5LBC7065V) were from Sigma Aldrich (USA), and native β-cyclodextrin was from Wacker (Munich, Germany) (purity ≥97%, humidity 12–14%). Chitosan (CS) was a donated sample (90% deacetylation degree, measured by NMR). Phenolphthalein, methylene blue and 1-naphthol (≥99%) were obtained from Merck (Darmstadt, Germany), and indigo carmine and methyl orange were from Panreac (Barcelona, Spain). For the synthesis process, sodium phosphate dibasic anhydrous (Na_2_HPO_4_) (≥98%) was bought from Sigma-Aldrich (St. Louis, MO, USA), sodium hydrogen carbonate (99.7%) from Panreac (Barcelona, Spain) and potassium pyrophosphate (97%) from Aldrich (St. Louis, MO, USA). All of these reagents were used as received.

### 4.2. Methods

#### 4.2.1. Synthesis Procedure

Citric acid (1.30 g) and sodium phosphate dibasic (0.28 g) are crushed with a polysaccharide (1.435 g) and β-cyclodextrin (1.435 g), then the mixture is heated up in an oven for times between 10 and 120 min, and temperatures between 140 and 180 °C. After crushing, the samples are washed using 100 mL of deionized water for 20 h and vacuum filtrated. The resulting liquid is dialyzed twice (24 h each time) with of 3.4 kDa membranes (Spectrum^TM^) and freeze-dried over a day. The solid is washed again with deionized water and dried in an oven at 102 °C. The resulting solid matrices are finally pulverized in a Retsch MM300 ball mill for 30 s.

#### 4.2.2. Characterization of the Crosslinked Networks

Infrared spectroscopy. Measurements of all the networks were obtained by the IRAffinity-1S instrument (Shimadzu, Kyoto, Japan), coupled with a Golden Gate^TM^ ATR device (SPECAC, Orpington, UK) and the LabSolution-IR software from Shimadzu. Infrared spectra were acquired between 4000 and 600 cm^−1^ with 32 scans and a resolution of 4 cm^−1^. The fittings of the curves were made with the tool present in Origin software “Multiple Peak Fit” (OriginLab, Northampton, MA, USA).

Colorimetry. The CIELAB parameters (lightness (L) from black to white and color (a*) from green to red and (b*) from blue to yellow) of the solid samples were analyzed using a CM-2300d Konica Minolta (Tokyo, Japan) spectrometer (SpectraMagic NX Lite software from Konica Minolta).

Fluorescence spectroscopy. Two milliliters of a solution of 5 ppm of 1-naphthol were placed in a cuvette and measured using an Edinburgh fluorimeter FLS920 (Kirkton Campus, UK). The fluorescence titration experiments were carried out as follows. After measuring the pure 1-naphthol sample, 100 µL of a solution of 1-naphthol at 5 ppm mixed with 2 g L^−1^ of polysaccharide were added to the first one. After each successive addition of 100 µL, a fluorescent emission spectrum is measured with an excitation wavelength of 291 nm.

Size exclusion chromatography. The Waters HPLC system was equipped with a 600 controller, a 717 autosampler and two detectors: a 2414 refractive index detector and a 996 photodiode array detector.

Dynamic Light Scattering. Estimations of the hydrodynamic radii of the soluble polymer gel fractions were performed using a DynaPro device (Wyatt Technology Corp., Santa Barbara, CA, USA) and the results were analyzed with the Dynamics^TM^ software from Wyatt.

#### 4.2.3. Properties of the Crosslinked Matrices

Saponification of insoluble products. Samples of the dried resins (ca. 100 mg) were placed in Falcon tubes with 12 mL of Na_2_CO_3_ 1 M and stirred for 3 h in a rotary mixer. After centrifugation and extraction of the supernatant liquid, the samples were washed and stirred for ½ h, centrifuged (twice) and dried in an oven at 60 °C until constant weight.

Swelling capacities. In an Eppendorf microtube, 10 mg of matrix were placed with 1.5 mL of deionized water for 24 h (additional swelling experiments were performed using acetone and 1-octanol). Once the swelling equilibrium is reached, the sample is centrifuged (10,000 rpm., 5 min), and the supernatant liquid is carefully eliminated; the final swollen sample is weighed and compared to the dry sample to obtain the mass swelling ratio *q_w_*.

Sorption of 1-naphthol. Ten milligrams of each polymer are placed in an amber vial and 10 mL of a 1-naphthol solution (20 ppm or 200 ppm) are added and stirred for 5 h; then, the solution is filtered through a 0.1 µm PVDF membrane (Durapore^®^), and analyzed using HPLC (Agilent 1100; Phenomenex Luna C18 column; methanol/water 65/35; 1 mL/min).

Sorption of dyes. Ten milligrams of the selected networks were introduced in 10 mL of a methylene blue and methyl orange solution (10 ppm each). The samples were placed in a rotary tube mixer for 24 h and finally centrifuged during 10 min at 4500 rpm. The supernatant is then analyzed using UV-Vis spectroscopy (Cary 8454 instrument; Agilent Technologies, Santa Clara, CA, USA) and separated. The hydrogel matrices are dried at 60 °C for 24 h. Then, 10 mL of water are added and the release of the dyes is measured by the UV-Vis spectrometer as a function of time.

Determination of accessible cyclodextrins. A solution of 0.1 mol/L sodium hydrogen carbonate buffer adjusted to pH 10.5 with 6 mol/L of NaOH is used to dissolve 3.6 × 10^–5^ mol/L of phenolphthalein with 0.4 mL of ethanol/L of solution. For each resin, samples (between 2 and 10 mg) are placed in vials with 7 mL of the previous solution. After 24 h, the samples are centrifuged during 5 min at 10,000 rpm. The amount of phenolphthalein absorbed is quantified by UV spectroscopy (Cary 8454, Agilent Technologies, with ChemStation software from Agilent). 

## Supplementary Materials

The following are available online at https://www.mdpi.com/2310-2861/6/4/51/s1, Scheme S1: Chemical structure of the polysaccharides. Table S1: CIELAB parameters of crosslinked products. Table S2: RGB color parameters of samples. Table S3: Calculation of unreacted cyclodextrin by size exclusion chromatography. Figure S1: Yield of soluble and insoluble products of the crosslinking reaction prepared using different feed ratios. Figure S2: Yields of insoluble fractions of networks crosslinked using different catalysts. Figure S3: FTIR spectra of crosslinking products using different catalysts and different ratios of disodium phosphate. Figure S4 (a–f): Infrared spectra of the saponification residues of the resins crosslinked at different times and temperatures. Table S4: Swelling degrees in water, acetone and 1-octanol. Figure S5: Sorption of 1-naphthol in polysaccharides. Figure S6: Total absorption and release kinetics of methylene blue and methyl orange. Table S5: Curve fitting results for kinetic measurements. Table S6: Amount of methylene blue and methyl orange (% mass) absorbed. Figure S7: SEC results of soluble fractions obtained from LBG/CD and CS/CD. Table S7: Hydrodynamic radii of soluble crosslinked samples. Figure S8: Infrared spectra for the citrate-crosslinked soluble and insoluble networks. Fluorescence quenching investigation for the soluble polysaccharides. Table S8: Interaction constants with 1-naphthol, obtained from fluorescence emission spectra. Figure S9: Fluorescence ratios as a function of the polysaccharide concentration.
