# Supplementary material for: Solventless Crosslinking of Chitosan, Xanthan, and Locust Bean Gum Networks Functionalized with β-Cyclodextrin
Source: Gels. 2020 Dec 15;6(4):51. doi: 10.3390/gels6040051 (PMC7768548; doi:10.3390/gels6040051)
Supplement: Supplementary file 1 [file gels-06-00051-s001.pdf]

gels

MDPI

SUPPLEMENTARY MATERIALS

Solventless synthesis of chitosan, xanthan and locust bean gum networks functionalized with β-cyclodextrin

*Max Petitjean, Florian Aussant, Ainara Vergara, and José Ramón Isasi**

Department of Chemistry. University of Navarra. 31080 Pamplona, Spain.

E-mail address: jrisasi@unav.es Tel.: +34 948 425 600; fax: +34 948 425 740

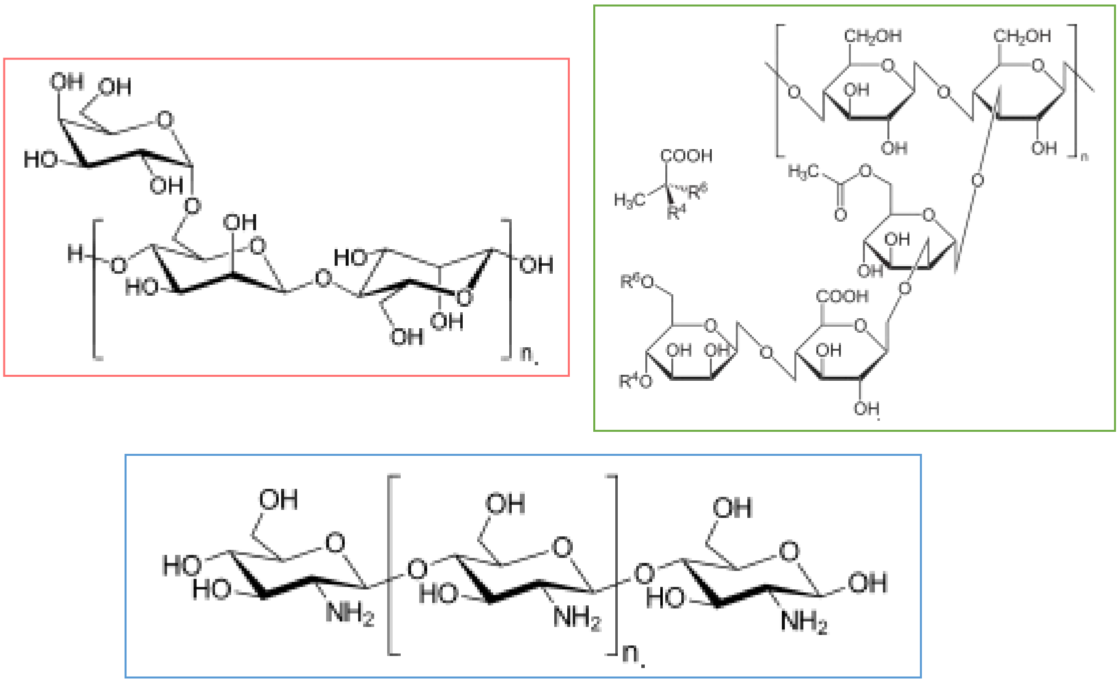

Scheme S1. Chemical structure of the three polysaccharides used in this work. LBG: locust bean gum (above, left), XG: xanthan gum (above, right), CS: chitosan (bottom).

Table S1. CIELAB parameters of xanthan gum (XG, locust bean gun (LBG) and chitosan (CD) crosslinked for 20 minutes at temperatures (T) between 140 and 170 °C and for times (t) between 10 and 120 minutes, at 170 °C.

SAMPLES	L*	A*	B*
**XG T170**	88.06	+0.67	+23.89
**XG T180**	86.81	+1.47	+26.17
**XG T45**	83.07	+2.02	+27.47
**XG T60**	81.81	+2.27	+27.20
**XG T120**	80.51	+2.88	+28.02
**LBG T140**	90.54	+0.47	+9.42
**LBG T150**	90.50	+0.68	+10.97
**LBG T160**	88.14	+0.89	+20.44
**LBG T170**	86.09	+1.57	+29.65
**LBG T180**	82.59	+2.73	+33.45
**LBG T10**	88.65	+0.95	+14.17
**LBG T45**	79.41	+3.25	+32.13
**LBG T60**	77.42	+3.83	+32.30
**LBG T120**	75.95	+4.30	+31.29
**CS T140**	85.48	+2.29	+28.13
**CS T150**	83.06	+3.02	+27.72
**CS T160**	75.44	+5.24	+29.44
**CS T170**	74.95	+4.94	+25.93
**CS T180**	73.19	+5.07	+25.71
**CS T10**	85.45	+2.06	+26.56
**CS T45**	72.07	+5.04	+25.19
**CS T60**	71.58	+5.20	+26.05
**CS T120**	67.72	+5.63	+24.87

Table S2. RGB color parameters of samples in Table S1.

RGB	T140	T150	T160	T170	T180	t10	t45	t60	t120
XG	-	-	-			-			
LBG									
CS									

Calculation of unreacted cyclodextrin by Size Exclusion Chromatography:

$$\%BCD_{nr} = 100 * \frac{V_t A}{mBCD_u V_{cr} A_0} [\beta CD]_0$$

where A_0 is the area of the peak (897600 a.u.) for a concentration $[\beta CD]_0$ of 1. 5mg·mL^{-1}, V_t is the total volume (mL) of the soluble fraction, V_{cr} is volume (0.1 mL) analyzed in the SEC experiment and $mBCD_u$ (1.6402 g) is the mass of βCD (with 12.4% of water) used for the synthesis reaction.

Table S3. Calculation of unreacted β-cyclodextrin (%bCD$_{nr}$) by SEC analysis

Name	Area	%bCD$_{nr}$	Name	Area	%bCD$_{nr}$
**CS$_{100}$**	0	0.0	**LBG$_{33}$ – βCD$_{67}$**	1038199	18.0
**CS$_{67}$ – βCD$_{33}$**	1020093	19.8	**XG$_{100}$**	0	0.0
**CS$_{50}$ – βCD$_{50}$**	1409638	28.7	**XG$_{67}$ – βCD$_{33}$**	803351	20.5
**CS$_{33}$ – βCD$_{67}$**	925072	18.9	**XG$_{50}$ – βCD$_{50}$**	1175151	26.3
**LBG$_{100}$**	0	0.0	**XG$_{33}$ – βCD$_{67}$**	1290698	22.4
**LBG$_{67}$ – βCD$_{33}$**	286758	4.67	**βCD$_{100}$**	2795547	57.0
**LBG$_{50}$ – βCD$_{50}$**	1190766	19.4			

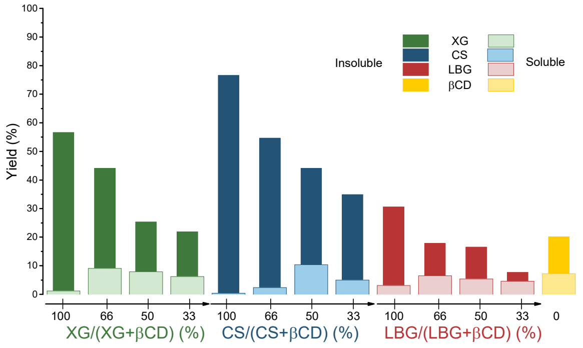

Figure S1. Yield of soluble and insoluble products of the crosslinking reaction of xanthan gum, locust bean gum and chitosan with β-CD and citric acid prepared using different feed (wt.) ratios: 1:0, 2:1, 1:1, 1:2, 0:1.

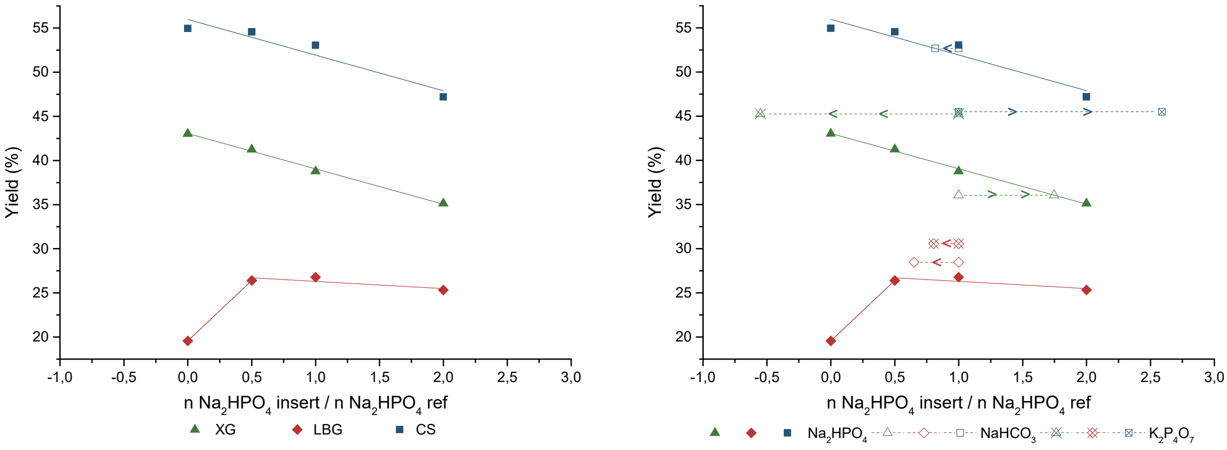

Figure S2. Yields of insoluble fractions of networks crosslinked using different catalysts (sodium phosphate dibasic, Na_2HPO_4, sodium hydrogen carbonate, $NaHCO_3$, and potassium pyrophosphate, $K_2P_4O_7$). The horizontal lines (right figure) for the polymers made with $NaHCO_3$ and $K_2P_4O_7$ represent the equivalent amount of Na_2HPO_4 needed to reach this yield.

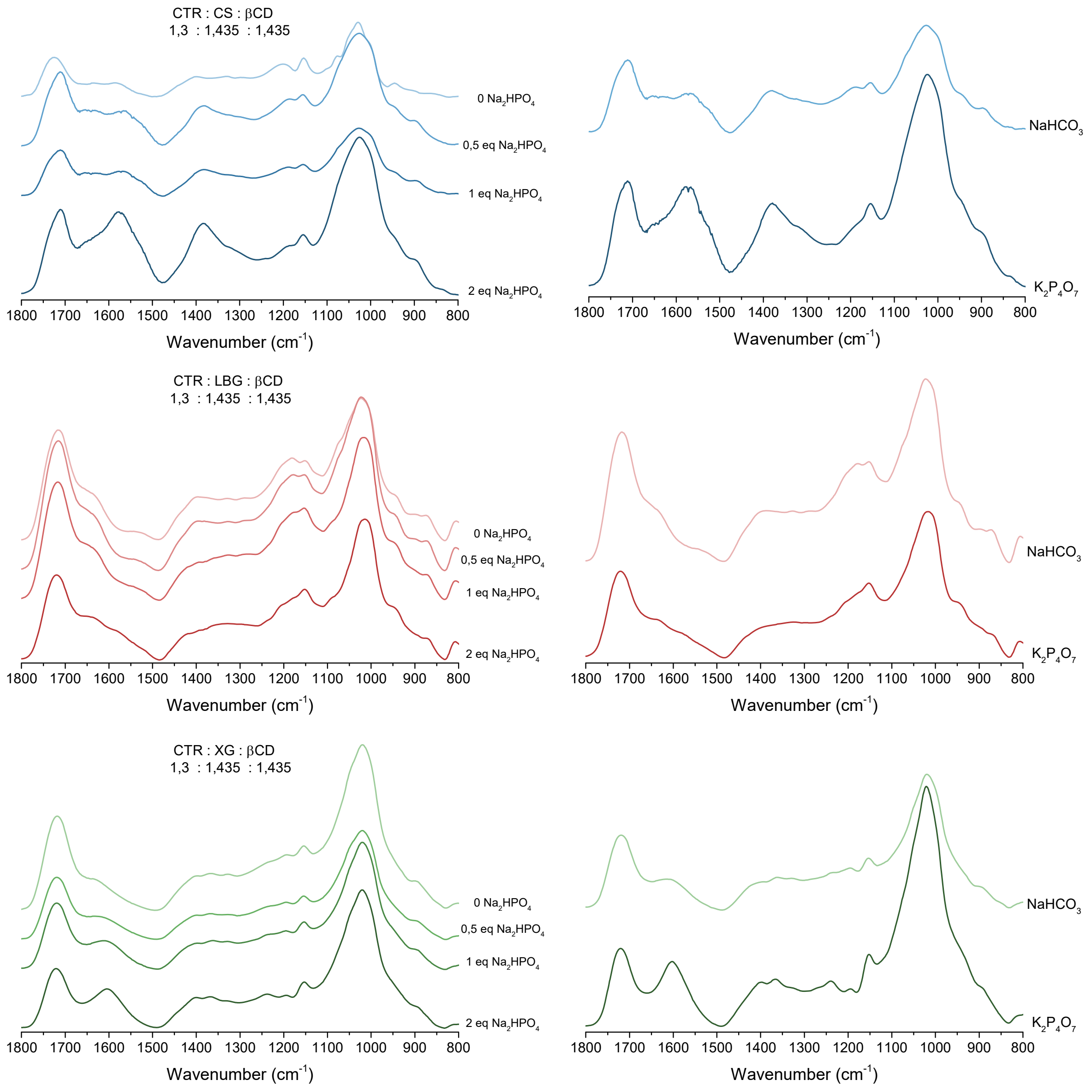

Figure S3. FTIR spectra in the 1800-800 cm^{-1} region of crosslinking products of citric acid (CTR), β-cyclodextrin (βCD) and the polysaccharides xanthan gum (XG), locust bean gum (LBG) and chitosan (CS), using different catalysts (right) and different ratios of disodium phosphate (left). For the xanthan samples, networks with a large catalyst ratio present a higher proportion of carboxylate groups (1600 cm^{-1}). For the chitosan networks, the amount of catalyst induces more crosslinking between amine and other groups (i.e. Maillard reactions with sugar carbonyls and amide bridges with citric acid). By comparison of the three different catalysts (Figure S2, right), $NaHCO_3$ produces similar structures to those obtained using a lower quantity of Na_2HPO_4, and the opposite occurs with $K_4P_2O_7$.

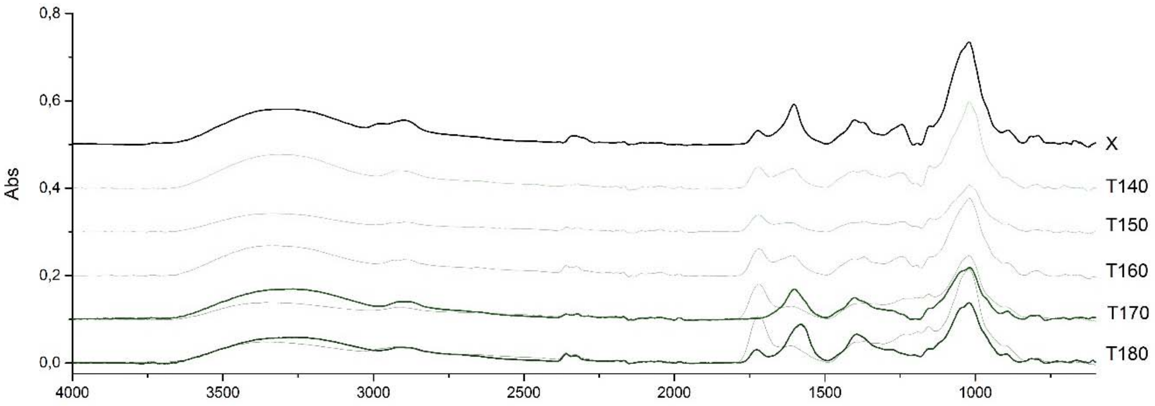

Figure S4a. Infrared spectra of xanthan gum (X) and the saponification residues of the resins crosslinked at different temperatures (*T* in °C) for 20 min. Blurred spectra correspond to the polymers prior to saponification.

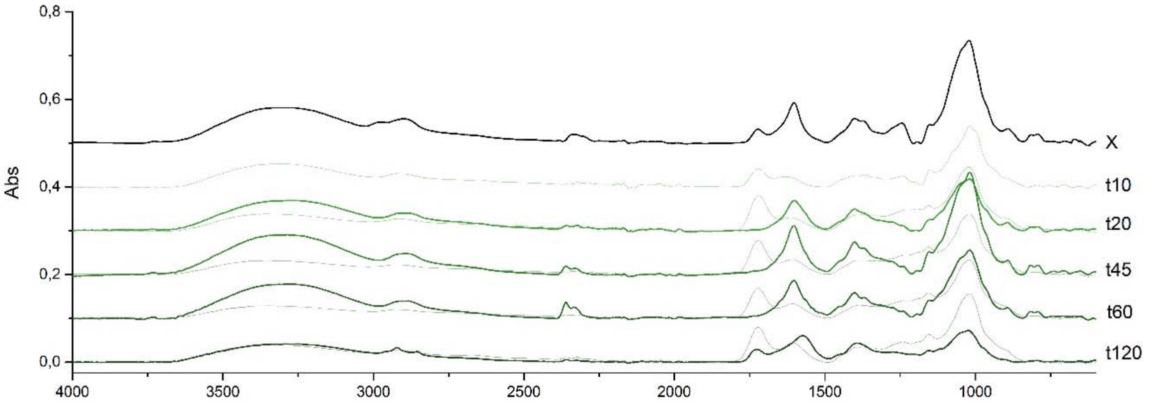

Figure S4b. Infrared spectra of xanthan gum (X) and the saponification residues of the resins crosslinked for different times (*t* in min) at 170 °C. Blurred spectra correspond to the polymers prior to saponification.

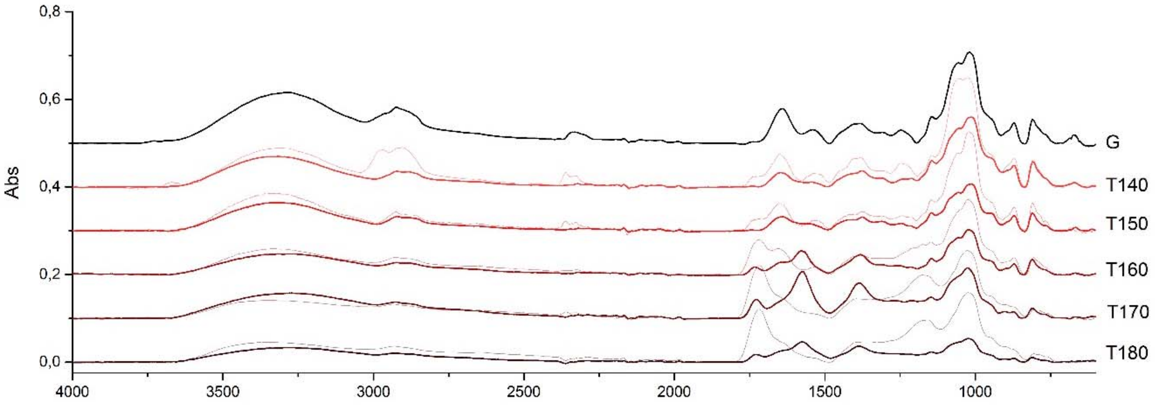

Figure S4c. Infrared spectra of locust bean gum (G) and the saponification residues of the resins crosslinked at different temperatures (*T* in °C) for 20 min. Blurred spectra correspond to the polymers prior to saponification.

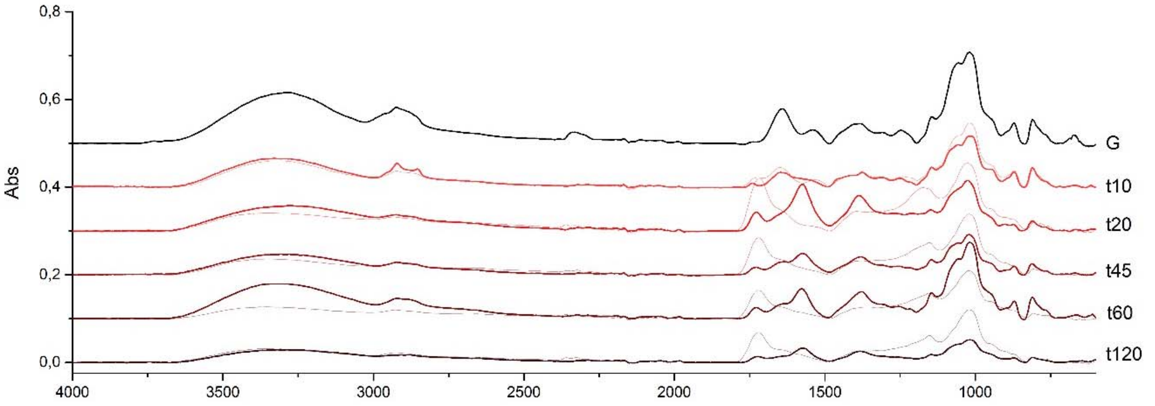

Figure S4d. Infrared spectra of locust bean gum (G) and the saponification residues of the resins crosslinked at 170 °C for different times (*t* in min). Blurred spectra correspond to the polymers prior to saponification.

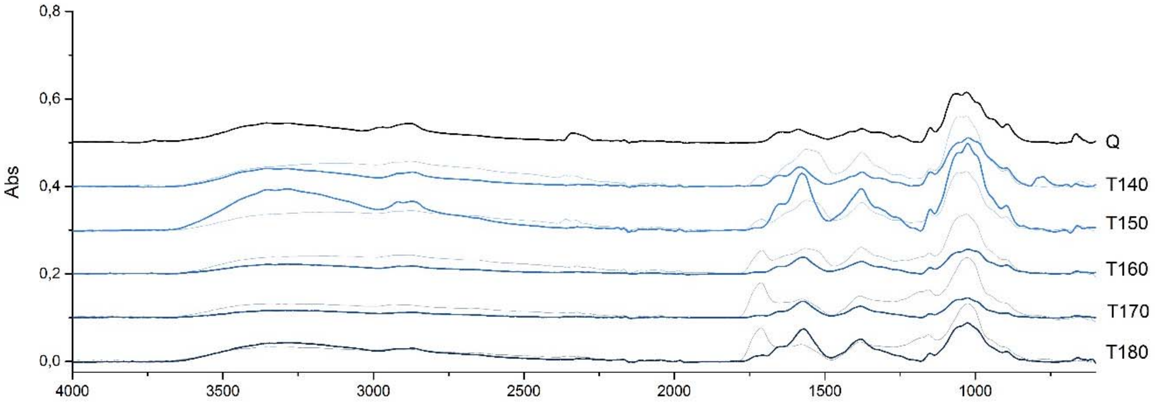

Figure S4e. Infrared spectra of chitosan (Q) and the saponification residues of the resins crosslinked at different temperatures (*T* in °C) for 20 minutes. Blurred spectra correspond to the polymers prior to saponification.

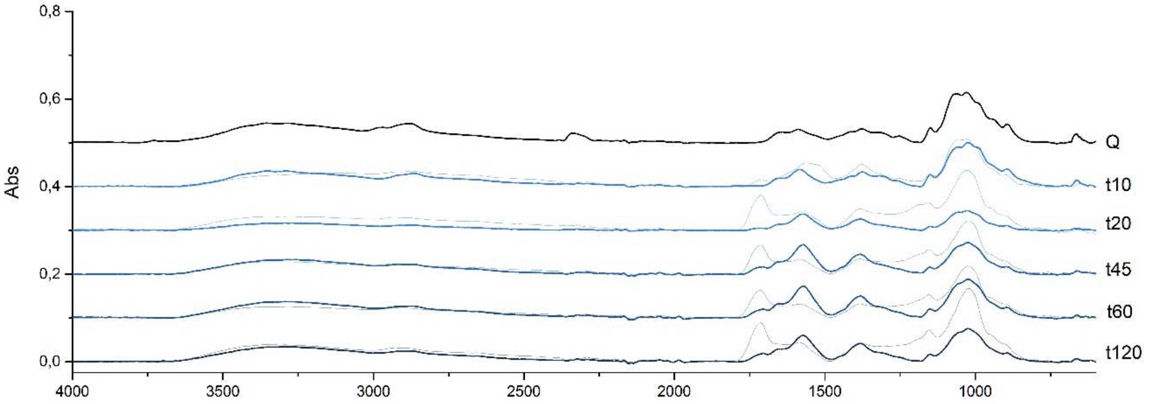

Figure S4f. Infrared spectra of chitosan (Q) and the saponification residues of the resins crosslinked at 170 °C for different times (*t* in min). Blurred spectra correspond to the polymers prior to saponification.

Table S4. Swelling degrees (q_w) of xanthan gum (XG), locust bean gum (LBG), chitosan (CS) and cyclodextrin crosslinked resins in water, acetone and 1-octanol.

Swelling Degree	CTR-XG	CTR-LBG	CTR-CS	CTR-βCD
Water	16.9	7.2	5.7	3.5
Acetone	1.0	1.1	1.0	1.2
1-Octanol	1.2	1.3	1.1	1.5

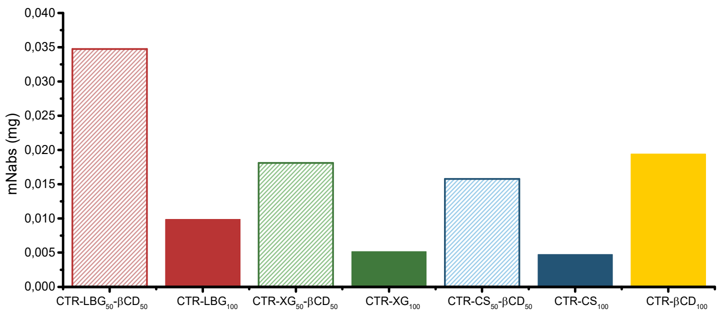

Figure S5. Sorption of 1-naphthol (mg) in polysaccharide (LBG, XG or CS) crosslinked with or without β-cyclodextrin (βCD) using citric acid (CTR).

Table S5. Correlation coefficient values (R^2) for the fitting of kinetic curves in Figure 5 using "pseudo first order" and "pseudo second order" equations.

Sample	t20	t10	t120	T140	T180
R^2 PO1	0.95314	0.9832	0.94366	0.91246	0.96966
R^2 PO2	0.99539	0.99999	0.99386	0.99988	0.9967

Table S6. Amount of methylene blue and methyl orange (% mass) absorbed by resins prepared with chitosan (CS), xanthan (XG), locust bean gum (LBG) and β-cyclodextrin (βCD) by crosslinking with citric acid (CTR).

m_{abs}/m_{ini} (%)	CTR-CS	CTR-XG	CTR-LBG	CTR-βCD
Methylene blue	90.1	88.1	84.8	88.8
Methyl orange	22.9	7.1	10.1	12.9

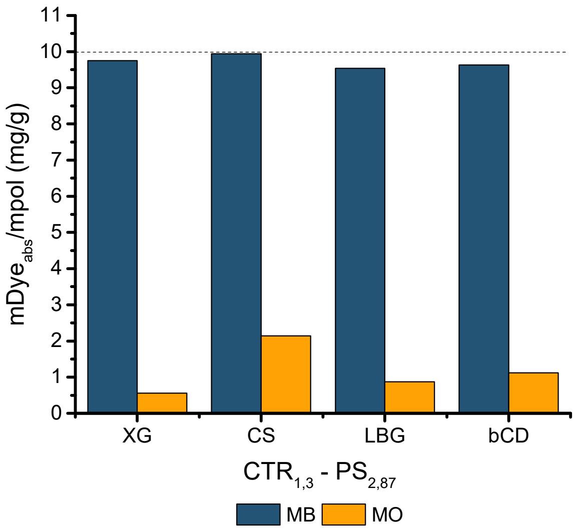

Figure S6a. Simultaneous absorption (mg per g of sample) of methylene blue (MB) and methyl orange (MO) in four matrices (xanthan, chitosan, locust bean gum and cyclodextrin, from left to right) prepared using the same mass ratio of CTR/polysaccharide (1.3/2.87).

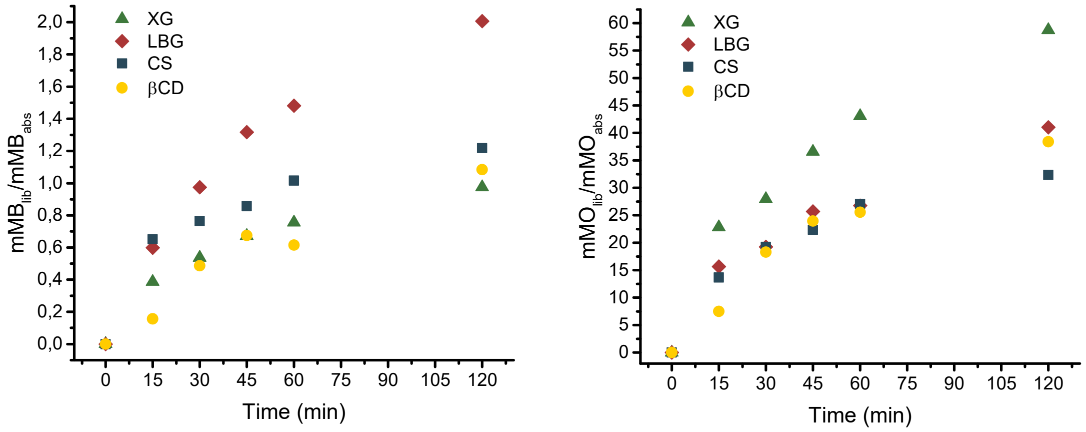

Figure S6b. Simultaneous release (relative to the amount absorbed, in %) of methylene blue (MB) and methyl orange (MO) from samples shown in Figure S6a.

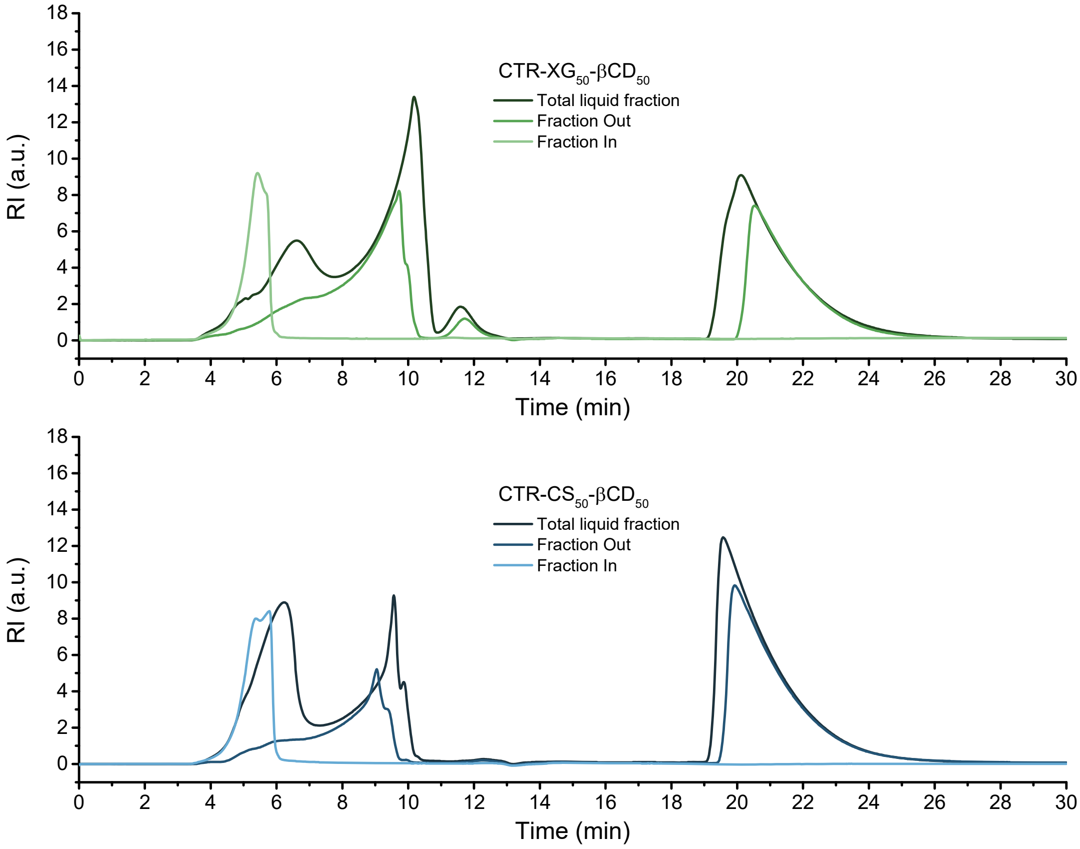

Figure S7. SEC results of soluble fractions obtained from LBG/CD (top) and CS/CD (bottom) (1:1 ratio) reaction products: total liquid fraction before dialysis, dialyzed liquid ("fraction out") and high molecular weight fraction retained inside the 3.5 kDa dialysis membranes ("fraction in").

Table S7. Hydrodynamic radii (R±ΔR, nm) at 20 °C and 37 °C of soluble crosslinked samples of cyclodextrin (BC100), locust bean gum (Gxxx), chitosan (Qyyy), and xanthan gum (Xzzz) prepared with different polysaccharide percentages (xxx%) measured by dynamic light scattering.

Sample	R (20 °C) nm	ΔR (20 °C) nm	R (37 °C) nm	ΔR (37 °C) nm
BC100	693	1136	247	179
G033	18190	38456	3050	4345
G050	14690	31573	7874	12850
G067	12428	20859	9755	13819
G100	54	27	800	831
Q033	317	261	203	63
Q050	319	278	169	81
Q067	13208	33123	1784	2611
Q100	459	442	428	479
X033	224	201	139	83
X050	997	1115	356	274
X067	348	411	137	85
X100	3891	7132	1857	2791

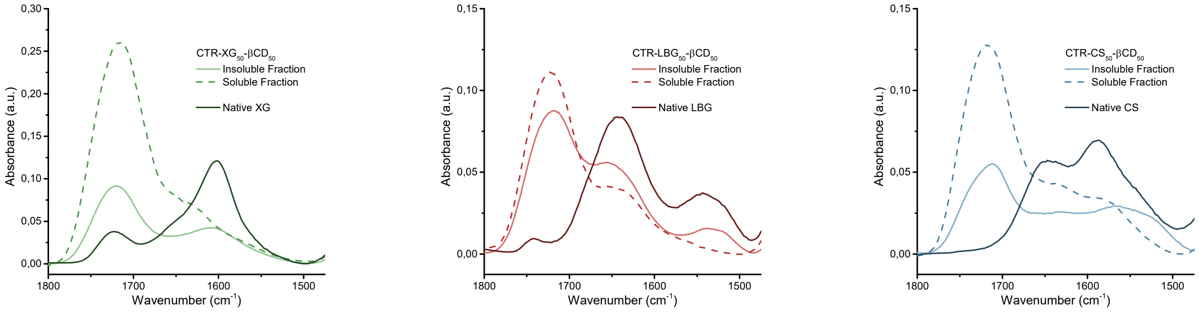

Figure S8. Infrared spectra in the 1800-1500 cm^{-1} region for citrate-crosslinked soluble and insoluble networks prepared with XG (left), locust bean gum (center) and chitosan (right) with βCD (1:1 ratio).

Fluorescence quenching investigation for the soluble polysaccharides

The changes detected in the UV-vis spectra of 1-naphthol in the presence of the soluble polysaccharide (PS) networks were too small to calculate interaction constants (results not shown), so fluorescence spectroscopy, a more sensitive technique, was used instead. The fluorescence intensity characteristic of 1-naphthol can be considered as the sum of the contributions of its free and associated forms. The interaction constants (K_i) can be calculated by means of a fluorescence quenching titration experiment if both contributions (i.e. their fluorescence constants k_s and k_{11}) are different. Thus, the total fluorescence (in arbitrary units) of a given sample is:

$$F = k_s[N] + k_{11}[PS:N]$$

where [N] and [PS:N] are the concentrations of the free and associated fluorophore, respectively. If a simple association model between 1-naphthol and the macromolecules (PS) are assumed, then the association constants K_i can be calculated as $K_i = [PS:N]/([PS]\cdot[N])$

The titration curves corresponding to the ratio of fluorescence of samples in the presence of polymers (F) and that in the absence of polymer (F_0) as a function of the PS concentration are:

$$\frac{F_0}{F} = \frac{1 + K[PS]}{\left(k_s/_{k_0}\right) + \left(k_{11}/_{k_0}\right)K[PS]}$$

Taking into account that k_s=k_0, then a non-linear equation is obtained:

$$\frac{F_0}{F} = \frac{1 + K[PS]}{1 + \left(k_{11}/_{k_0}\right)K[PS]}$$

In the special case that we have a non-fluorescent complex (k_{11}=0), an affine function is deduced:

$$\frac{F_0}{F} = 1 + K[PS]$$

The fluorescence intensity results (see Figure S9) show that, for all the samples tested, there is a quenching in the fluorescence emission of 1-naphthol, measured at 289 nm. The fluorescence spectra of the pure unreacted polysaccharides were compared with those for polysaccharide/β-cyclodextrin soluble networks prepared using a 1:2 ratio of the two. The fluorescence quenching effect is more important in pure chitosan and smaller in the case of both LBG samples tested (pure LBG and the LBG/CD soluble crosslinked polymer), analogously to the effects observed in the preliminary UV-vis spectra. Nevertheless, the interaction equilibrium constants, which is the parameter of interest here, show a different trend.

Table S8. Interaction constants (Kex (289 nm) b>0) with 1-naphthol, obtained from fluorescence emission spectra (excitation at 289 nm), and the corresponding *b* values for the pure polysaccharides (chitosan in 1% acetic acid, locust bean gum and xanthan gum), crosslinked cyclodextrin (BC100) and the crosslinked resins with 33% of polysaccharide. Interaction constants with the hypothesis of a non-fluorescent complex (b=0), and those obtained using the excitation spectra (Kem 471 nm) are shown for comparison purposes.

Sample	Kem (471nm)	Kex (289nm) (b=0)	Kex (289nm) b>0	b
CS pure	0.4535	0.4806	0.4289	-0.0958
LBG pure	0.0750	0.0431	2.8618	0.9634
XG pure	0.1815	0.1452	1.2621	0.8221
BC100	0.1800	0.1519	0.9491	0.7775
Q033 (CS)	0.2308	0.1522	0.9682	0.7801
G033 (LBG)	0.0992	0.1026	1.1193	0.8672
X033 (XG)	0.2019	0.2316	0.3936	0.3661

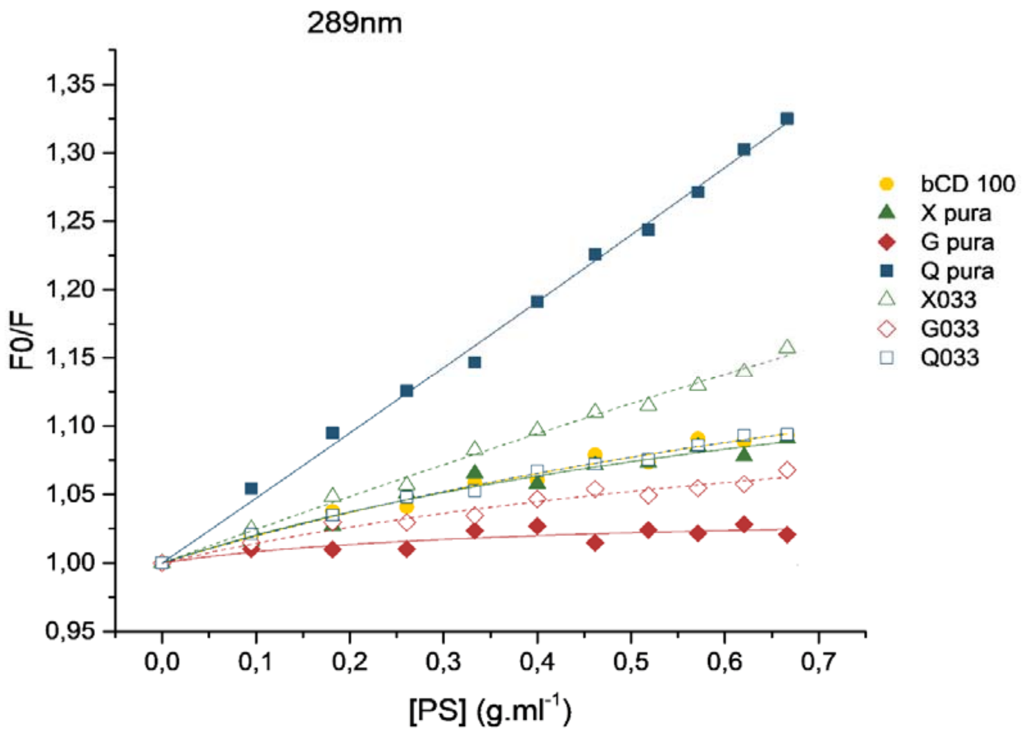

Figure S9. Fluorescence ratios F_0/F as a function of the polysaccharide concentration, in g/L (see Table S8).
